# Rapid and Non-destructive Detection and Identification of Two Strains of *Wolbachia* in *Aedes aegypti* by Near-Infrared Spectroscopy

**DOI:** 10.1371/journal.pntd.0004759

**Published:** 2016-06-30

**Authors:** Maggy T. Sikulu-Lord, Marta F. Maia, Masabho P. Milali, Michael Henry, Gustav Mkandawile, Elise A. Kho, Robert A. Wirtz, Leon E. Hugo, Floyd E. Dowell, Gregor J. Devine

**Affiliations:** 1 Mosquito Control Laboratory, QIMR Berghofer Medical Research Institute, Brisbane, Queensland, Australia; 2 Environmental Health and Ecological Sciences Thematic Group, Ifakara Health Institute, Ifakara, United Republic of Tanzania; 3 Centers for Disease Control and Prevention, Atlanta, Georgia, United States of America; 4 Stored Product Insect and Engineering Research Unit, United States Department of Agriculture/Agricultural Research Services, Center for Grain and Animal Health Research, Manhattan, Kansas, United States of America; Mahidol University, THAILAND

## Abstract

The release of *Wolbachia* infected mosquitoes is likely to form a key component of disease control strategies in the near future. We investigated the potential of using near-infrared spectroscopy (NIRS) to simultaneously detect and identify two strains of *Wolbachia pipientis* (*w*MelPop and *w*Mel) in male and female laboratory-reared *Aedes aegypti* mosquitoes. Our aim is to find faster, cheaper alternatives for monitoring those releases than the molecular diagnostic techniques that are currently in use. Our findings indicate that NIRS can differentiate females and males infected with *w*MelPop from uninfected wild type samples with an accuracy of 96% (N = 299) and 87.5% (N = 377), respectively. Similarly, females and males infected with *w*Mel were differentiated from uninfected wild type samples with accuracies of 92% (N = 352) and 89% (N = 444). NIRS could differentiate *w*MelPop and *w*Mel transinfected females with an accuracy of 96.6% (N = 442) and males with an accuracy of 84.5% (N = 443). This non-destructive technique is faster than the standard polymerase chain reaction diagnostic techniques. After the purchase of a NIRS spectrometer, the technique requires little sample processing and does not consume any reagents.

## Author Summary

Near infrared spectroscopy (NIRS) is a technique that measures specific frequencies of light absorbed by C-H, O-H, S-H and N-H functional groups. Mosquito samples are grouped based upon absorption differences between their chemical properties. In this study, we used NIRS to differentiate 1) *Aedes aegypti* infected with either of the two strains of intracellular bacterium *Wolbachia* (*w*Mel and *w*MelPop) from wild type *Ae*. *aegypti* and 2) *Aedes aegypti* infected with *w*Mel from those infected with *w*MelPoP. NIRS facilitated the differentiation of *w*Mel and *w*MelPop from wild type samples and samples infected with either of the *Wolbachia* infected strains with high prediction accuracies over their lifespan. Predictive models were derived from initial calibration data sets and validated against independent cohorts. Prediction accuracies were high (82–98%) regardless of the cohort mosquitoes were sampled from. The results show that NIRS may have real potential as an alternative method for monitoring *Wolbachia* incidence in mosquitoes. A rapid, simple and cost-effective surveillance tool suitable for resource-poor areas and large urban release programs would be of great utility for evaluating *Wolbachia-*based interventions. The models developed during this study require further validation using field collections.

## Introduction

The mosquito *Aedes aegypti* is the primary vector of the four human dengue, chikungunya and Zika viruses. Options for developing effective vaccines or chemotherapeutics are limited [1] and vector control remains fundamental for the prevention of disease transmission. One of the most promising strategies exploits the maternally-transmitted intracellular bacteria, *Wolbachia pipientis*. *Wolbachia* are naturally found in up to 60% of all insect species and are propagated through insect populations via reproductive manipulations. In some insect species such as mosquitoes [2,3], tsetse flies[4] and *Liriomyza trifolii* [5] a phenomenon called cytoplasmic incompatibility (CI) mediated by *Wolbachia*, modifies the sperm of infected males such that crosses with uninfected females do not produce viable offsprings. The effect is rescued in crosses with infected females, leading to a reproductive advantage that favors *Wolbachia* transmission through the population. CI and phenotypes associated with *Wolbachia* infection can interrupt mosquito-borne pathogen transmission cycles. Transinfection of the *Wolbachia-*free dengue vector *Ae*. *aegypti* with the virulent *Wolbachia* strain *w*MelPop was associated with a 50% reduction in mosquito life span and 100% CI [2]. Furthermore, *Wolbachia* infection interferes with subsequent infections by a diverse range of pathogens, including dengue and chikungunya viruses, *Plasmodium gallinaceum*, and filarial worms [6–8]. The mechanisms of pathogen interference are yet to be fully defined. *Wolbachia* infection can stimulate the host insect immune system, leading to the "immune priming" a hypothesis of pathogen interference [7–11]. However, this hypothesis does not hold in all situations [12,13]. Alternatively, competition for nutrient resources required by *Wolbachia* and pathogens, particularly cholesterol, appears to be a major factor contributing to dengue virus (DENV) blocking by *Wolbachia* [14,15].

To date, two *W*. *pipientis strains* have been successfully transinfected into *Ae*. *aegypti*; the virulent strain *w*MelPop [2] and the relatively benign strain *w*Mel [16]. In the first demonstration of the capacity of *Wolbachia* to reduce the risk of dengue transmission under field settings, *w*Mel infected *Ae*. *aegypti* were released into suburbs of Cairns, Australia, harboring endemic populations of the vector. Subsequent monitoring indicated the successful invasion and stable establishment of the bacteria in the *Ae*. *aegypti* population [17]. Reduced DENV vector competence of field-collected *Ae*. *aegypti* subsequent to that invasion has been demonstrated [18] and a program of field evaluation studies and pilot releases is now occurring globally[19,20]. Future rearing and release programs will need careful monitoring to track the establishment, patterns and stability of *Wolbachia* invasions.

Currently, molecular techniques, such as polymerase chain reaction (PCR), are used to detect *Wolbachia* infections in mosquitoes [21–23] but there is concern that their cost and technical complexity are not amenable to the evaluation of large-scale, programmatic interventions in the developing world [24,25].

Near-infrared spectroscopy (NIRS) is a non-destructive and almost instantaneous technique that allows high throughput differentiation of biological samples. Mosquito samples are grouped based upon absorption differences in the NIR region that result from differences in the composition and concentration of organic molecules. The technique does not require reagents and involves minimal sample processing (up to 15 seconds per sample). Collection of NIR spectra from a mosquito requires only a single, 3 second interrogation. Once a calibration model has been developed and validated, prediction of independent samples takes a few seconds. Parameters such as age and species identity can be predicted from the same spectrum. It has been estimated that as an age grading and species identification tool, NIRS is 35 times cheaper and over 16 times faster than PCR and conventional microscopy techniques [26].

Previously, NIRS has been used to differentiate the morphologically identical African malaria vectors; *Anopheles gambiae* and *Anopheles arabiensis* with an accuracy of 80–90%. NIRS has also been used to categorise individuals within these species into age categories (> or < 7 d old); that are more relevant to the probability of *Plasmodium* infection. NIRS grouped mosquitoes into these categories with an accuracy of 78–90% respectively [26–33].

In this study we examined the potential of using NIRS as a high throughput technique for detecting the presence or absence of *Wolbachia* in laboratory-reared *Ae*. *aegypti* mosquitoes. NIRS has previously been successfully applied to discriminate species and strains of bacteria [34–36] and recently to detect *Wolbachia* in fruit flies [37]. This study represents the first use of NIRS to detect the presence or absence of *Wolbachia* in male and female *Ae*. *aegypti*.

## Materials and Methods

### Rearing and collection of mosquitoes

We compared wild type *Ae*. *aegypti* (from a colony established from *Wolbachia-*free material collected from Cairns in Jan 2015), *Ae*. *aegypti* transinfected with *w*Mel (from a colony established from *Wolbachia* release suburbs in Cairns April 2015) and the PGYP1 strain of *Ae*. *aegypti* transinfected with *w*MelPop and subsequently out crossed to wild type *Ae*. *aegypti* from Cairns (supplied courtesy of the Eliminate Dengue group, Monash). All strains were reared at the insectary of QIMR Berghofer Medical Research Institute, Australia, in separate rooms under identical conditions; 27°C, 70% humidity, 12:12 hr day:night lighting. Larvae were fed on Tetramin tropical flakes (Tetra Melle, Germany). Pupae were transferred into cages measuring 40 × 40 × 30 cm for adult emergence. Adults were fed on 10% sugar solution daily and blood fed on a human volunteer for 15 min every 7 d according to human research ethics protocol (QIMR HREC980).

Each of the three *Ae*. *aegypti* mosquito strains (*w*Mel transinfected, *w*MelPop transinfected and wild type) were represented by two separate cohorts: one was used to develop calibration and validate models and the other was used to test the models. Adults from the calibration cohort were collected at 1, 5, 10, 15, 19 and 20 d post emergence. This was to ensure that the calibration model was applicable across a wide range of age groups. For the validation set, adults were collected 5 d post emergence. Samples were stored in RNA*later* for 2–4 weeks before scanning by a NIR spectrometer [28]. PCR of the wsp gene was used to confirm presence or absence of *Wolbachia*-infection in *Ae*. *aegypti* colonies [23]. The *w*Mel and *w*MelPop colonies used in these experiments were 100% *Wolbachia* infected due to the PCR amplification of the wsp gene from 100% of tested specimens (N = 39 and 40, respectively) whereas the wild type *Ae*. *aegypti* colony was completely uninfected (N = 47).

### Scanning of mosquitoes

**Fig 1:**
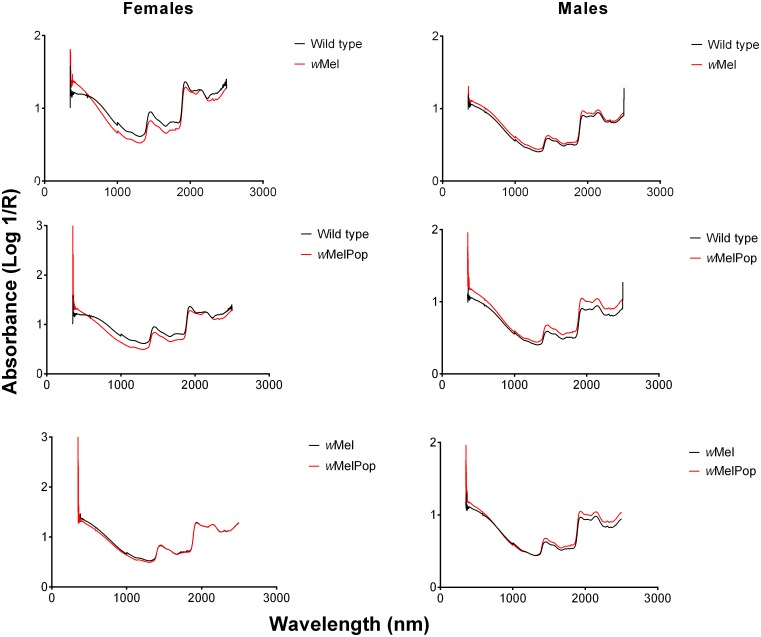
Examples of average spectra collected from heads and thoraces of *Wolbachia* infected and wild type male and female *Ae*. *aegypti* mosquitoes.

All mosquitoes collected were transferred to the Ifakara Health Institute, Tanzania for scanning using a LabSpec 5000 NIR spectrometer (ASD Inc, Boulder, CO) according to established protocols [27]. Prior to scanning, residual RNA*later* was removed by blotting mosquito specimens with a filter paper. Examples of average spectra collected from heads and thoraces of individual mosquitoes are shown in Fig 1.

### Data analysis

Model development and calibration was conducted as previously published [27]. In brief, spectra between 500–2350 nm were analyzed using partial least square (PLS) regression in GRAMS Plus/IQ software (Thermo Galactic, Salem, NH). Calibrations were developed to differentiate: 1) *w*MelPop infected females from wild type females, 2) *w*Mel infected females from wild type females 3) *w*MelPop infected males from wild type males, 4) *w*Mel infected males from wild type males 5) *w*MelPop infected females from *w*Mel infected females and 6) *w*MelPop infected males from *w*Mel infected males. For each data set, at least 144 mosquitoes were used to develop calibration models.

**Fig 2:**
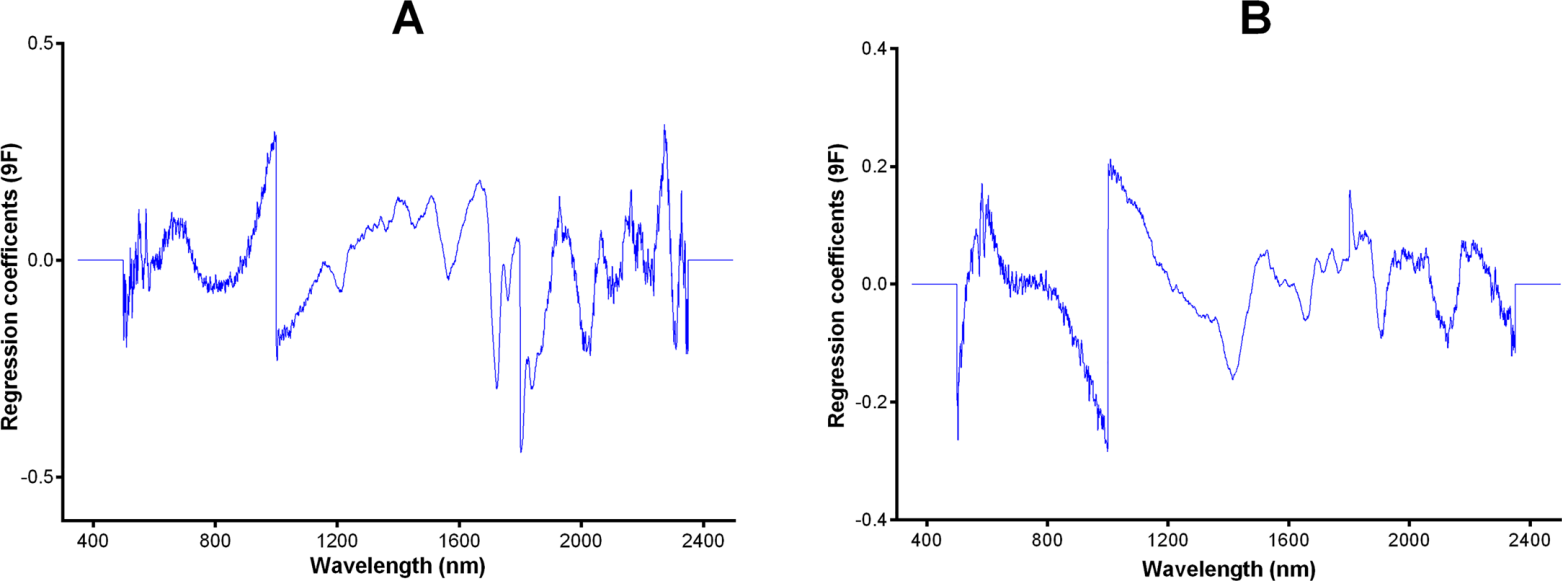
Regression coefficient plots used to predict the presence or absence of infection. They are based on 9 PLS regression factors. The plots show peaks that influenced the differentiation of female *w*MelPop from female *w*Mel (panel A) and female *w*Mel from female wild type *Ae*. *aegypti* (panel B).

In the PLS model, a value of “1” was assigned to wild type samples whereas a value of “2” was assigned to *Wolbachia* infected mosquitoes. Similarly, to differentiate male and female mosquitoes infected with *w*Mel from those infected with *w*MelPop, a value of “1” was assigned to *w*Mel and a value of “2” was assigned to *w*MelPop infected mosquitoes. A value of 1.5 was considered the threshold for correct or incorrect classification. For example, all wild type samples predicted above the 1.5 cut-off point were considered misclassified and vice versa. These calibration models were then applied to independent data sets (mosquitoes from cohort 1 that were not used to develop the model and mosquitoes from cohort 2 to test whether they could predict: 1) the presence or absence of *w*Mel *or w*MelPop in female and male *Ae*. *aegypti* mosquitoes, and 2) to differentiate between *w*MelPop and *w*Mel infections. Regression coefficient plots used to differentiate *w*MelPop from *w*Mel infected female *Ae*. *aegypti* and *w*Mel infected from wild type *Ae*. *aegypti* using 9 factors in the PLS model are shown in Fig 2.

A logistic regression analysis was undertaken in SPSS version 22.0 (SPSS Inc. Chicago) to determine the effects of age, sex, cohort and infection type on the prediction accuracy of NIRS. Backward stepwise procedure was used to control for potential confounding factors between covariates (removal criteria, *P* > 0.10; re-entry criteria, *P* ≤ 0.05). Non-linear relationships were examined through scatter plots. Results are expressed as adjusted odds ratios and 95% confidence intervals. Where quoted, statistical significance is at the conventional *P* < 0.05 level.

## Results and Discussion

**Fig 3:**
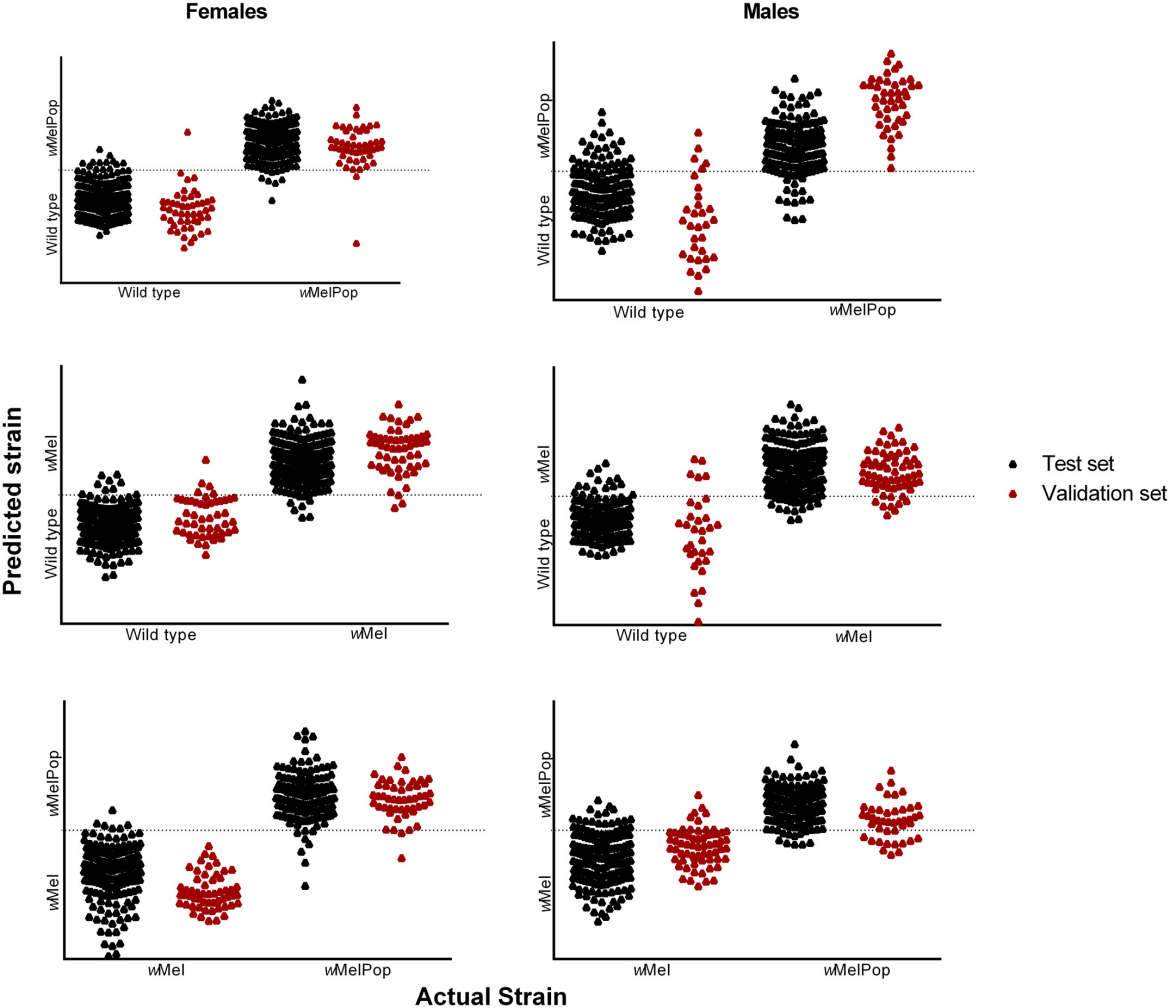
NIRS differentiation of *Wolbachia* infected and wild type male and female *Ae*. *aegypti* mosquitoes using validation and test samples. The dotted line indicates the classification cut off point as predicted by the NIRS.

We demonstrated that NIRS has considerable potential as a high throughput tool for differentiating *Wolbachia* infected and uninfected mosquitoes. Using PLS models developed from a cohort of mosquitoes of various ages, NIRS could detect the presence or absence of specific *Wolbachia* types in independent mosquito collections. Regardless of their age category and the specific cohort, females and males transinfected with *w*MelPop were differentiated from wild types with an average accuracy of 96% (N = 299) and 87.5% (N = 377) respectively. Similarly, NIRS differentiated between *w*Mel and wild type females with an average accuracy of 92% (N = 352) whereas infected males were differentiated from uninfected males with an accuracy of 88.5% (N = 444). NIRS was also able to categorise different strains of *Wolbachia*; female and male *Ae*. *aegypti* mosquitoes infected with *w*MelPop could be differentiated from *w*Mel infected mosquitoes with an accuracy of 96.6% (N = 442) and 84.5% (N = 443), respectively. The overall accuracy for each cohort is presented in Table 1 and Fig 3.

**Table 1:** Percentage accuracy of *Wolbachia* detection using cross validation and prediction analyses.

Infection type	% Accuracy [N] Cross validation^1^	% Accuracy [N] Validation set^2^	% Accuracy [N] Test set^3^
*w*MelPop ♀/ wild type ♀	92 [259]	97 [200]	95 [99]
*w*Mel ♀/ wild type ♀	89 [256]	91 [248]	93 [104]
*w*MelPop ♂/ wild type ♂	85 [144]	82 [301]	93 [76]
*w*Mel ♂/ wild type ♂	91 [160]	89 [346]	88 [98]
*w*MelPop ♀/ *w*Mel ♀	95 [200]	95 [337]	98 [105]
*w*MelPop ♂/ *w*Mel ♂	89 [177]	90 [335]	79 [108]

^1^ Accuracy of mosquitoes used to develop calibration models

^2^ Accuracy of cohort 1 mosquitoes that were used to validate calibration models

^3^ Accuracy of cohort 2 mosquitoes that were used to test calibration models

Logistic regression analysis indicated that age (*P* < 0.001), the interaction term between age and infection type (*P* < 0.001), sex (*P* = 0.046) and infection type (*P* < 0.001) significantly affected the prediction accuracy of NIRS. Odds of an accurate prediction of the presence or absence of an infection increased 1.089 fold (95% CI: 1.039–1.142) from younger (1 d old) to older (20 d old) mosquitoes. Compared to wild type *Ae*. *aegypti*, the odds of NIRS accurately predicting the presence of *w*MelPop changes 0.919 fold (95% CI: 0.865–0.976) and 0.862 fold (95% CI: 0.815–0.911) for *w*Mel from younger to older age groups. NIRS was 1.647 times (95% CI: 1.008–2.691) more accurate at predicting infection in females than males. NIRS was 4.321 times (95% CI: 2.447–7.629) more accurate in predicting *w*Mel infection, and 2.318 times (95% CI: 1.303–4.123) more accurate in predicting the presence of *w*MelPop relative to wild type mosquitoes.

We conducted this study under the assumption that there was a difference in the cellular constituents of *Wolbachia* transinfected and wild type mosquitoes. It was also hypothesized that those differences would be reflected in the heads and thoraces of the mosquitoes hence producing unique NIR spectra that could be used for their identification.

Spectral regions between 700–2350 nm have previously been reported to be specific for bacteria differentiation and these regions have been successful at detecting and identifying different species of bacteria in an isolated system [34,36] as well as for differentiation of different strains of bacteria [38] using NIRS. We have also shown in this study that bands at the1400-2350 nm spectral regions were responsible for differentiating wild type from *Wolbachia* infected *Ae*. *aegypti* and sharp bands at 2000–2350 nm might have been responsible for differentiating *Ae*. *aegypti* transinfected with different strains of *Wolbachia*. In particular, bands at 2000-2300nm spectral band region suggest aliphatic C-H and methylene stretching might have largely contributed to the differentiation of *w*Mel from *w*MelPop infected mosquitoes (Fig 2A) whereas sharp bands between 1400-1700nm related to C-H first overtone of amide band combinations and aromatic groups largely contributed to differentiation of *Wolbachia* infected from uninfected mosquitoes [39] (Fig 2B).

Infections with *w*MelPop, and to a lesser extent, *w*Mel can achieve high densities in a broad range of mosquito tissues [16]. Recent metabolic analyses demonstrate that *Wolbachia* utilize and modulate cellular levels of nutrients including cholesterol [14], amino acids [15], proteins carbohydrates [40] and glycogen [41]. It is possible that NIRS differentiates wild type from *Wolbachia* infected mosquitoes through spectral signals generated from qualitative and quantitative differences in these nutrients. Alternatively, differences in bacteria density may also differentiate *w*Mel and *w*MelPop. There is very limited genomic variation between *w*MelPop and *w*Mel, with the exception of a large inversion, triplication of copy number of a 19 kb region [42,43] and insertion of an IS5 element into gene *WD1310* [44]. The triplication was lost during subsequent cell passaging and mosquito infection to generate the *w*MelPop PGYP strain used in these experiments. Currently, quantitative PCR (qPCR) of a region of the IS5 element is used to differentiate between *w*Mel and *w*MelPop strains [45]. The ability to distinguish *w*Mel or *w*MelPop using our rapid and non-destructive technique could introduce large time and cost savings over conventional PCR techniques.

The calibration model was developed using a range of adult ages (age is known to affect the absorbed and reflected spectra in mosquitoes) successfully predicted the *Wolbachia* type in single age cohorts (Table 2). NIRS predictions were generally more accurate for female mosquitoes than for males. The proportion of female individuals that were misclassified was 3–7% whereas the proportion of misclassified males was 7–12% and this difference was significant (*P* = 0.046). Stronger NIRS signals from females may be due to higher *Wolbachia* densities in females relative to males. This has been observed for *Wolbachia* strains *w*AlbA and *w*AlbB within *Aedes albopictus* [46] and may reflect higher *Wolbachia* densities in mosquito ovaries [16,47]. When differentiating *w*Mel or *w*MelPop infected females from wild type females, 1 d old female mosquitoes contributed to the highest misclassification (Table 2). This might be explained by the presence of under-developed ovaries in 1 d old mosquitoes relative to older mosquitoes [47].

**Table 2:** Percentage accuracy with which pairs were differentiated at various ages (1–20 days), and the specific identification accuracy for the individual components of those pairs.

Infection type	N	1d	5d	10d	15d	19d	20d	Specific [N] for first member of pair	Specific [N] for second member of pair
*w*MelPop ♀ / wild type ♀	299	87	98	100	97	100	96	95 [151]	97 [148]
*w*Mel ♀ / wild type ♀	352	85	86	92	95	84	96	93 [208]	87 [144]
*w*MelPop ♂ / wild type ♂	377	70	87	87	89	80	92	89 [197]	79 [180]
*w*Mel ♂ / wild type ♂	444	90	93	100	81	76	95	90 [262]	89 [182]
*w*MelPop ♀ / *w*Mel♀	442	95	99	98	95	92	97	95 [199]	97 [243]
*w*MelPop ♂ / *w*Mel ♂	443	98	97	98	75	79	90	89 [197]	86 [246]

*Wolbachia-*mediated disease control strategies may take a number of different forms in the future. The currently established model is a replacement strategy in which a relatively benign strain such as *w*Mel is driven into wild mosquitoes through unidirectional CI. The mosquito population that develop from these interactions are *Wolbachia*-infected, fit and stable but less capable of transmitting virus [17].

The *w*MelPop strain of *Wolbachia* is known for its virulent nature. Cells infected with *w*MelPop have been reported to cause increased cell apoptosis and reduced survival in adult mosquitoes [2]. This affects fitness of *Ae*. *aegypti* and prevents the establishment of infections in field populations, limiting its current usefulness for biological control. However, its virulence may be exploited by a proposal to first drive *w*MelPop into a target wild type population and then “crash” that population through the poor fitness of the resulting phenotype [48].

Another possibility is to use unidirectional CI to implement variations on the sterile insect technique (SIT). In one scenario, male-only releases of *Wolbachia*-infected mosquitoes could be used to overwhelm mating interactions with wild type females, a strategy referred to as the incompatible insect technique (IIT) [49,50]. Unlike conventional SIT, the object of IIT for *Aedes* species would be to suppress local mosquito populations below the threshold required for effective disease transmission. In that scenario, it is crucial that no *Wolbachia*-infected females are released (which could lead to population replacement instead of population suppression). Therefore, a proposed strategy is to combine conventional SIT with IIT, which would remove the risk of vector population replacement [50,51]. Proof-of-concept studies have shown the viability of combining irradiation and *Wolbachia*-based approaches [51–54]. Depending on the virus-blocking capability of the introduced *Wolbachia* strain, this combined approach might also have the additional benefit of eliminating the risk of virus transmission by those released females. Alternatively, bidirectional CI where no mating with *Wolbachia* infected mosquitoes are viable could also be used to overwhelm and replace the local population [55]. In all of these instances it will be essential to monitor the infection status of the release colonies and the stability and spread of infection population suppression strategies. NIRS may present a rapid means of making these assessments.

Our examination of over 900 male and female wild type *Ae*. *aegypti* mosquitoes or *Ae*. *aegypti* transinfected with either *w*Mel or *w*MelPop demonstrated that NIRS has potential as a high throughput tool to identify *Wolbachia* infection across a range of mosquito age. NIRS offers demonstrable improvements over technically demanding, expensive and time consuming molecular assays. PCR techniques are suited to limited surveillance using small or pooled samples that may not represent the true spatial or temporal heterogeneity of extensive field populations. The high throughput PCR assay recently described by Lee and colleagues for detecting *Wolbachia* in dengue mosquitoes requires costly DNA extraction kits and PCR reagents [22,23]. Unpublished analysis of cost using a local supplier (Qiagen Pty LTD, Victoria, Australia) on 09/07/2015 demonstrated that a single sample processed using this high throughput PCR-based technique would cost approximately 6 USD after the initial outlay for a real time PCR machine with an option for high resolution melt analysis. Comparatively, NIRS can collect a diagnostic spectrum from an individual mosquito within 3 seconds.The NIRS data that result from a single interrogation can be used to predict more than one characteristic in each individual e.g. age and species in the *An*. *gambiae* complex [26–28]. No reagents or sample-specific preparations are needed, hundreds of individual mosquitoes can be scanned in a day and results can be analysed and reported upon immediately. After the initial cost of the spectrometer (Ca. 60,000 USD), only minimal labor related costs are required to run the NIRS technique. The >80% accuracy observed in this study is sufficient to rapidly assess success or failure of *Wolbachia* based control interventions such as those targeting dengue and other emerging infectious diseases such as Zika. If NIRS can also be used to predict the age of dengue and Zika vectors, it would define many key factors associated with vectorial capacity and the evaluation of disease transmission risk. The use of NIRS to measure a number of key disease vector characteristics has now been demonstrated in several studies [26–28]. NIRS promises to improve the speed and capacity of program evaluations and risk mapping exercises, especially in remote regions with little infra-structure or in dense urban environments with very high surveillance costs.
